# Simultaneous evaluation of physical and social environmental correlates of physical activity in adults: A systematic review

**DOI:** 10.1016/j.ssmph.2017.05.008

**Published:** 2017-05-15

**Authors:** Alexia Sawyer, Marcella Ucci, Russell Jones, Lee Smith, Abi Fisher

**Affiliations:** a Health Behaviour Research Centre, Department of Epidemiology and Public Health, University College London, Gower Street, London WC1E 6BT, UK; b UCL Institute for Environmental Design and Engineering, The Bartlett Faculty of the Built Environment, Central House, University College London, 14 Upper Woburn Place, London WC1H 0NN, UK; c Glasgow Centre for Population Health, The Olympia Building, University of Glasgow, Glasgow G12 8QQ, UK; d The Cambridge Centre for Sport and Exercise Sciences, Dept. of Life Sciences, Anglia Ruskin University, UK

**Keywords:** Active living, Built environment, Social capital, Neighbourhood

## Abstract

**Background:**

Ecological models of physical activity posit that social and physical environmental features exert independent and interactive influences on physical activity, but previous research has focussed on independent influences. This systematic review aimed to synthesise the literature investigating how features of neighbourhood physical and social environments are associated with physical activity when both levels of influence are simultaneously considered, and to assess progress in the exploration of interactive effects of social and physical environmental correlates on physical activity.

**Methods:**

A systematic literature search was conducted in February 2016. Articles were included if they used an adult (≥15 years) sample, simultaneously considered at least one physical and one social environmental characteristic in a single statistical model, used self-reported or objectively-measured physical activity as a primary outcome, reported findings from quantitative, observational analyses and were published in a peer-reviewed journal. Combined measures including social and physical environment items were excluded as they didn’t permit investigation of independent and interactive social and physical effects. Forty-six studies were identified.

**Results:**

An inconsistent evidence base for independent environmental correlates of physical activity was revealed, with some support for specific physical and social environment correlates. Most studies found significant associations between physical activity and both physical and social environmental variables. There was preliminary evidence that physical and social environmental variables had interactive effects on activity, although only 4 studies examined interactive effects.

**Conclusions:**

Inconsistent evidence of independent associations between environmental variables and physical activity could be partly due to unmeasured effect modification (e.g. interactive effects) creating unaccounted variance in relationships between the environment and activity. Results supported multiple levels of environmental influence on physical activity. It is recommended that further research uses simultaneous or interaction analyses to gain insight into complex relationships between neighbourhood social and physical environments and physical activity, as there is currently limited research in this area.

## 1 Introduction

Despite several health benefits of regular participation in physical activity (Ekelund et al., 2015, Reiner et al., 2013), most individuals living in industrialised nations lead insufficiently active lifestyles (Hallal et al., 2012). Interventions that target individuals have had limited success (Hillsdon, Foster, & Thorogood, 2005), perhaps partly because individual-level correlates are estimated to explain only 20–40% of reported variance in physical activity (Spence & Lee, 2003). Research and policy has therefore increasingly adopted a broader, ecological approach to activity which considers a combination of individual, social, physical, cultural and political correlates.

Systematic reviews of the literature have identified some consistent physical environment correlates of physical activity, including land use mix, connectivity and residential density which all have positive associations with activity (McCormack and Shiell, 2011, Saelens and Handy, 2008). Access to green space may also be important: a study including over 200,000 adults reported cross-sectional associations between green space access and increased self-reported walking and moderate-to-vigorous physical activity (MVPA) (Astell-Burt, Feng, & Kolt, 2014).

The social environment has also been examined in relation to physical activity. In particular, cognitive and structural social capital constructs have been explored, encompassing aspects of perceived or objective social cohesion, trust, social support, safety, social participation and social resources (e.g. collective efficacy to enforce normative behaviours and reciprocity in sharing personal resources) (Moore & Kawachi, 2017). In a recent systematic review of 38 studies, Samuel, Commodore-Mensah, & Himmelfarb (2014) identified several characteristics of the social environment associated with overall physical activity, walking and sports participation, with higher quality social environments (i.e. increased sense of community, trustworthiness, reciprocity, social cohesion and social control) indicating higher levels of activity. There is also some evidence for a negative association between physical activity and crime and a positive relationship between physical activity and perceived safety, although findings are inconsistent. Several reasons could contribute to inconsistent results: i) inadequate measurement of crime resulting in measurement error, ii) use of physical activity outcomes that are not neighbourhood-based and therefore may have weaker relationships with the neighbourhood environment and iii) lack of consideration of features of the physical and social environment that may mediate or moderate the effects under investigation (Foster & Giles-Corti, 2008).

A core tenet of ecological models of physical activity is that correlates are embedded in a complex system whereby multiple environmental and individual characteristics are interrelated and exert independent and interactive effects (Sallis et al., 2006). While a growing literature examines independent effects of environmental correlates, there has been very little focus on their interactive or synergistic effects on physical activity despite empirical and theoretical evidence of interplay between social and physical environments (e.g. social interaction is related to structural elements including provision of communal space (Yancey, 1971), physical disorder is associated with collective efficacy (Sampson & Raudenbush, 1999) and bidirectional reciprocal associations existing between social and physical disorder as purported by broken windows theory (Keizer, Lindenberg & Steg, 2008)). The scientific value of examining social and physical effects simultaneously (rather than only controlling for other environmental correlates) is to explore the concurrent influences of social and physical environmental features on physical activity, as hypothesised in ecological models.

Conceptualising concurrent influences could elucidate counter-intuitive relationships between the environment and physical activity. For example, although there is an established relationship between area deprivation and poorer health outcomes and behaviours, including physical activity (Ecob & Macintyre, 2000), a study in two Scottish neighbourhoods found that the deprived neighbourhood had more recreation centres, sport centres and street cleaning than the affluent neighbourhood, undermining the assumption that more deprived areas would be physically less supportive of activity (Macintyre, Maciver & Sooman, 1993). Various studies in Europe, USA and Australia also report that physical activity resources are not fewer in more deprived areas (Cradock et al., 2005, Giles-Corti and Donovan, 2002, Van Lenthe et al., 2005). In Canada and USA, lower levels of physical activity were self-reported in areas that are objectively-classified as highly walkable (according to physical metrics like connectivity) than in less walkable areas (Jack and McCormack, 2014, King, 2008). In such instances, features of the social environment or micro-scale features of the physical environment may modify the impact of physical walkability metrics.

Broader understanding of pathways of influence could also inform intervention development. A walking intervention involving the installation of walking route signage and leadership for local walking groups in two low-income neighbourhoods in Ireland had only a marginal effect on physical activity (Burgoyne, Coleman, & Perry, 2007). Reasons behind the null effect were examined in a qualitative study (n=53), finding that social barriers such as anti-social behaviour persisted following the intervention (Burgoyne et al., 2007). This highlights the necessity of simultaneous observation of social and physical environmental correlates of activity to develop effective interventions.

To our knowledge, there is no existing review of research which simultaneously examines social and physical environmental correlates of physical activity. As such, the purpose of this systematic review was to ask how physical and social environmental features are associated with physical activity when both levels of influence are simultaneously considered in statistical models, and to assess the extent to which these influences have been considered simultaneously and interactively in the literature. Simultaneous consideration of physical and social environments in statistical models could have taken different forms, for example variables could have been included in a mediation analysis or simultaneously included in a single multivariate regression model. In every instance, results for social and physical environmental variables had to have been reported and treated as target exposures (not confounders for which associations with activity were not tested or presented).

## 2 Material and methods

The review was designed in accordance with PRISMA guidelines. The quality of the studies included in the review was assessed using the quality appraisal tool considering the study’s research question, theoretical perspective, study design, context, sampling, data collection, data analysis, reflexivity concerning limitations, generalisability and ethics (Croucher, Myers, Jones, Ellaway & Beck, 2013). This tool has been used for related literature reviews (Croucher, Quilgars, Wallace, Baldwin & Mather, 2003). Studies were not included unless they met the ‘essential’ quality criteria.

### 2.1 Literature search

A systematic search of the literature was conducted on literature published until the end of February, 2016, using the scientific databases Embase, Ovid MEDLINE, PsycINFO and Social Policy and Practice. A reference search of relevant articles was also conducted to obtain any missing literature and original articles were identified from conference proceedings.

Search terms in Table 1 were used to access literature assessing related physical and social environment constructs and all physical activity outcomes. The social environment encompassed social capital constructs but did not encompass social composition constructs such as neighbourhood socioeconomic status (Moore & Kawachi, 2017). Social support and modelling of physical activity (e.g. seeing others being active) were not included as they are not typically included at the environmental level in ecological models for physical activity and such constructs could predominantly be a consequence of an environment that is conducive to physical activity. Search terms did not explicitly cover transport-related aspects of the physical environment (e.g. ‘access to transit’) or specific aspects of the urban form (e.g. ‘connectivity’) but it was expected that any such aspects would be identified through selected search terms.

**Table 1:** Search terms and syntax.

**Construct**	**Search terms**
Physical environment	(Built environment or physical environment or connectivity or walkab* or neighbourhood or neighbourhood or green space or greenspace or office or workplace or housing or gym or school or community centre or care home or nursing home or park or recreation* facility* or recreation* space) in abstract OR title
Social environment	(Social capital or social control or social* cohesi* or social network or trust or safety or crime or social environment or social interaction or socio-cultural) in abstract OR title.
Physical activity	(Physical activity or walk or sedentary or exercise* or sit* or active travel* or active transport*) in abstract or title

### 2.2 Eligibility criteria

Articles were included if they used an adult (≥15 years old) sample living in rural, suburban or urban environments in a developed country (or countries), simultaneously considered at least one physical and one social environmental characteristic in a single statistical model, used physical activity as a primary outcome, reported findings from quantitative, observational analyses and were published in a peer-reviewed academic journal after 1980. Clinical populations were excluded from the review. Combined measures including both social and physical environment items were excluded as they didn’t permit investigation of independent and interactive social and physical effects. There were very few studies in environments other than neighbourhoods (e.g. schools, workplaces); therefore, the review was limited to neighbourhoods.

[Anonymous] conducted the title and abstract reviews. [Anonymous] and [Anonymous] independently conducted the full-text review. Inter-rater reliability was 93%; disagreements at the full-text review were resolved through discussion.

### 2.3 Data extraction

Data extracted included author(s), year of publication, journal, sample characteristics (size, age, sex, country) and measurement tools. Results from univariate models were not always presented therefore it was not possible to compare univariate and multivariate models to assess whether variables retained or lost significance when entered into multivariate models.

## 3 Results

**Fig. 1:**
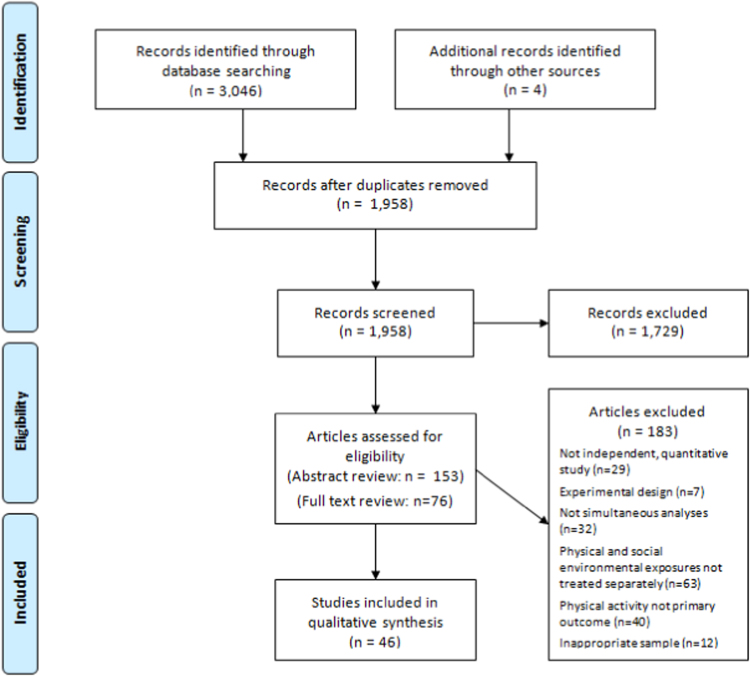
Flowchart depicting the stages of the search process and study selection.

Fig. 1 illustrates the study selection process from study identification to inclusion. The literature search obtained 3019 records. Title, abstract and full-text screening against inclusion criteria obtained 46 studies including 65 separate models which were included in a narrative review. A meta-analysis was deemed inappropriate due to the heterogeneity of exposures and outcomes. The combination of diverse exposures under one category in a meta-analysis could have produced an inappropriate summary (Higgins & Green, 2011). All studies met the required quality standard for inclusion.

Characteristics of each study are displayed in Table 2. Twenty-two studies were conducted in the USA. Thirty-seven studies used a male and female sample and 8 studies used an exclusively older adult sample (although the age range defined as ‘older adult’ varied from >60 years old to >66 years old). Sixteen studies used deprived samples. Study sample sizes ranged from n=148 to n=68,968; 17 studies had a sample size of n>3,000.

**Table 2:** Study characteristics.

First author, year	Sample	N	Country	Physical activity outcome	Social environment tool(s)	Physical environment tool(s)
Amorim, Azevedo & Hallal, 2010	Adults (20–69 years); urban	972	Brazil	Overall active travel, overall leisure-time; self-reported	Subjective	Subjective
Adlakha et al., 2015	Adults (21–65 years); urban	2015	USA	Overall; self-reported	Subjective	Subjective
Bird et al., 2009	Older adults (>60 years); urban	333	Australia	Overall; self-reported	Subjective	Subjective
Booth, Owen, Bauman, Clavisi & Leslie, 2000	Older adults (>60 years); urban	449	Australia	Overall; self-reported	Subjective	Subjective
Bracy et al., 2014^c^	Adults (20–65 years); older adults (>66 years); urban	2068; 718	USA	MVPA; accelerometer Walking active travel, walking leisure-time; self-reported	Subjective	Objective, subjective
Caspi et al., 2013	Adults (>18 years); urban	729	USA	Walking active travel, walking leisure-time; self-reported	Subjective	Objective
Cleland et al., 2010^c^	Women (18–45 years); urban/rural	4108	Australia	Overall leisure-time; self-reported	Subjective	Subjective
Eichinger et al., 2015	Adults (18–91 years); urban/rural	904	Austria	Overall, overall leisure-time, overall active travel; self-reported	Subjective	Subjective
Foster, Hillsdon & Thorogood, 2004^b^	Older adults (64–94 years); urban	582	USA	Walking^a^; self-reported	Subjective	Objective
Florindo et al., 2013^c^	Adults (>18 years); urban	890	Brazil	Overall; self-reported	Subjective	Subjective
Foster et al., 2004	Adults (16–74 years); urban/rural	4265	England	Walking; self-reported	Subjective	Subjective
Gomes et al., 2011	Adults (>18 years); urban	6166	Brazil	Walking leisure-time; self-reported	Subjective	Subjective
Granner, Sharpe, Hutto, Wilcox & Addy, 2007	Adults (>18 years); urban/rural	2025	USA	MVPA, walking; self-reported	Subjective	Subjective
Handy et al., 2008^c^	Adults; urban	1682	USA	MVPA^a^; self-reported	Subjective	Objective, subjective
Heesch, Giles-Corti & Turrell, 2014	Adults (40–65 years); urban	10,233	Australia	MVPA; self-reported	Subjective	Subjective
Huston, Evenson, Bors & Gizlice, 2003	Adults (>18 years); urban/rural	1701	USA	Overall leisure-time; self-reported	Subjective	Subjective
Jack & McCormack, 2014^c^	Adults (>18 years); urban	1875	Canada	Walking active travel^a^, walking leisure-time^a^; self-reported	Subjective	Objective, subjective
Jia et al., 2014^c^	Adults (15–75 years); urban	1582	China	Walking active travel, walking leisure-time; self-reported	Subjective	Subjective
Kamphuis et al., 2008^c^	Adults (25–75 years); urban	3839	Netherlands	MVPA; self-reported	Subjective	Subjective
Karusisi et al., 2012	Adults (30–79 years); urban	7105	France	MVPA^a^; self-reported	Subjective	Subjective
King et al., 2006^c^	Adults (18–85 years); urban	645	USA	Walking active travel, walking leisure-time, MVPA; self-reported	Subjective	Subjective
King, 2008	Older adults (>65 years); urban	190	USA	Overall^a^; self-reported	Subjective	Objective
Li & Fisher, 2004^c^	Older adults (>65 years); urban	582	USA	Overall^a^; self-reported	Subjective	Subjective
Lovasi et al., 2013	Adults (>18 years); urban	8034	USA	Overall active travel; self-reported	Objective	Objective
Mason et al., 2011^c^	Adults (>16 years); urban	5657	Scotland	Walking^a^; self-reported	Subjective	Objective, subjective
Poortinga, 2006	Adults (>16 years); urban/rural	14,836	England	Walking, MVPA, overall; self-reported	Subjective	Subjective
Prince et al., 2011^b^	Adults (>18 years); urban	3,383	Canada	Overall; self-reported	Objective, subjective	Objective
Prince et al., 2012	Adults (>18 years); urban	4727	Canada	Overall; self-reported	Objective	Objective
Salvador, Florindo, Reis & Costa, 2009^c^	Older men (>60 years); urban	152	Brazil	Overall; self-reported	Subjective	Subjective
Strath et al., 2012	Older Adults; urban	148	USA	Light, MVPA, overall; objective	Objective, subjective	Objective, subjective
Trumpeter & Wilson, 2013	Adults (>18 years); urban	290	USA	Walking leisure-time; self-reported	Subjective	Subjective
Troped, Tamura, Whitcomb & Laden, 2011	Women (40–59 years); urban/rural	68,968	USA	Walking, MVPA; self-reported	Subjective	Subjective
Van Dyck et al., 2013	Woman (18–46 years); urban/rural	4139	Australia	Walking leisure-time, walking active travel; self-reported	Subjective	Objective
Van Dyck et al., 2015	Adults (18–66 years); urban	7273	11 countries	MVPA; accelerometer	Subjective	Subjective
Van Lenthe et al., 2005^c^	Adults (20–69 years); urban	8,767	Netherlands	MVPA, overall active travel, overall leisure-time; self-reported	Objective	Objective
Voorhees & Rohm Young, 2003	Women (20–50 years); urban	285	USA	MVPA; self-reported	Subjective	Subjective
Wallmann, Bucksch & Froboese, 2012	Adults (18–65 years); urban	310	Germany	Walking, MVPA; self-reported	Subjective	Subjective
Weber Corseuil, Hallal, Corseuil, Schneider & d’Orsi, 2012^c^	Older adults (>60 years); urban	1656	Brazil	Overall leisure-time, overall active travel; self-reported	Subjective	Subjective
Wen et al., 2007	Adults (>18 years); urban/rural	41,545	USA	Walking; self-reported	Subjective	Subjective
Wen & Zhang, 2009^b^	Adults (>18 years); urban	3530	USA	MVPA; self-reported	Subjective	Objective
Wilbur, Chandler, Dancy & Lee, 2003b^c^	Women (20–50 years); urban	300	USA	MVPA; self-reported	Subjective	Subjective
Wilbur, Chandler, Dancy & Lee, 2003a	Women (20–50 years); urban	399	USA	MVPA; self-reported	Subjective	Subjective
Wilcox, Castro, King, Housemann & Brownson, 2000	Women (>40 years); urban/rural	2.338	USA	Overall; self-reported	Subjective	Subjective
Rohm Young & Voorhees, 2003	Women (20–50 years); urban	234	USA	MVPA; self-reported	Subjective	Subjective
Zhou et al., 2013	Adults; urban	478	China	MVPA, overall leisure-time, overall active travel; accelerometer, self-reported	Subjective	Subjective
Zoellner, Hill, Zynda, Sample & Yadrick, 2012	Adults (>18 years); urban	372	USA	Walking, overall; self-reported	Subjective	Subjective

a Neighbourhood-specific physical activity, N.B. Karusisi et al. (2012) studied location non-specific and neighbourhood-specific physical activity.

b Within-neighbourhood level results unavailable; between-neighbourhood results reported.

c Predominantly deprived sample. All objective measures of physical activity were accelerometry.

**Fig. 2:**
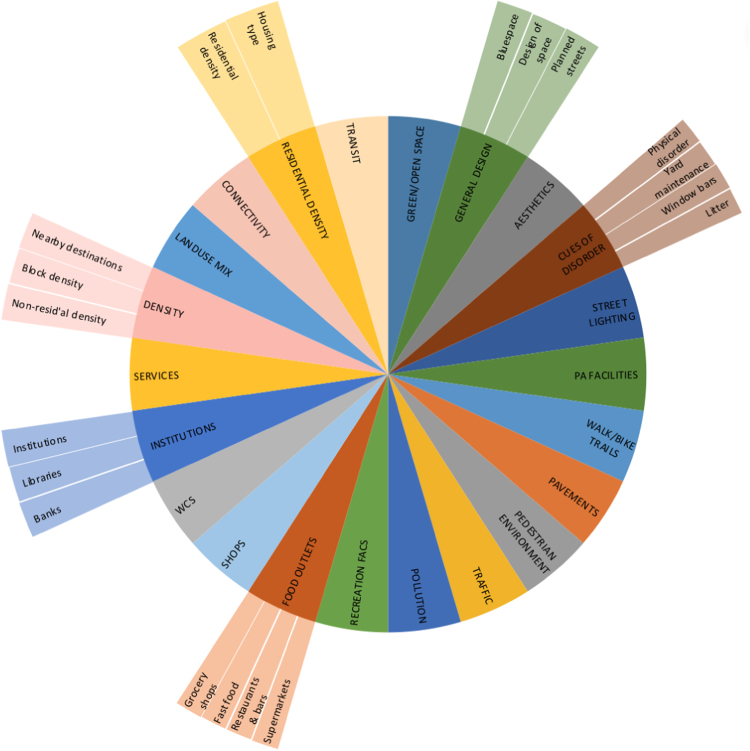
Physical environment variable clusters.

**Fig. 3:**
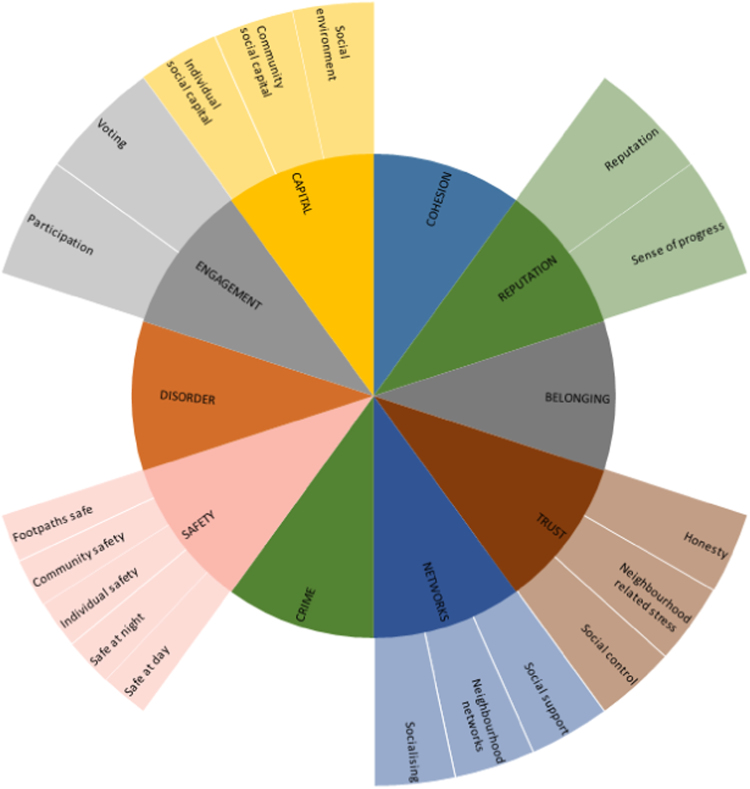
Social environment variable clusters.

Physical environment or social environment variables that were conceptually very similar (e.g. voting and participation, or housing density and housing type) were organised into clusters for illustrative purposes to aid interpretation of results (Fig. 2, Fig. 3). Physical variables that were used in more than 4 studies (i.e. approximately 10% of studies) were treated as an independent cluster (e.g. street lighting), with the exception of provision of WCs and pollution which did not form coherent clusters with other physical environment variables. Although there was some overlap between clusters, they were kept separate in order to retain a degree of specificity. There were more physical environment clusters due to i) inclusion of more physical environment variables in analyses and ii) wider use of conceptual models of social capital, collective efficacy and safety, encouraging broader use of formal terminology to organise social variables.

### 3.1 Independent physical environment correlates

Overall study results are reported in the Supplementary material (Tables S1 and S2). Where studies had conflicting results (e.g. results differed by sub-sample), this was demarcated and the key result reported. Where studies reported multiple physical activity outcomes (e.g. walking for transport, walking for leisure), these were reported individually.

When simultaneously accounting for aspects of the social environment, overall, there was inconsistent evidence that variables measuring communal space, street conditions or physical activity facilities were related to walking. Perceived access to services (e.g. stores, post offices, transit) were positively related to active travel by walking (Jack and McCormack, 2014, Jia et al., 2014) but there were inconsistent results for leisure-time walking (Jack and McCormack, 2014, Jia et al., 2014, Trumpeter and Wilson, 2014). Conversely, recreation facilities had inconsistent associations with general walking, active travel by walking, leisure-time walking and light physical activity (Supplementary material: Table S1). Land use mix had conflicting associations with self-reported active travel, leisure-time walking and objectively-measured light physical activity (King et al., 2006, Strath et al., 2012). Finally, greater connectivity was related to increased active travel by walking in 2 studies (Jack and McCormack, 2014, King et al., 2006), but had a null effect on leisure-time walking. When combined with non-residential density (access to different non-residential destinations), connectivity was related to active travel by walking by was negatively associated with leisure-time walking (Van Dyck, Veitch, De Bourdeaudhuij, Thornton, & Ball, 2013).

There were few consistent significant physical environmental correlates of MVPA except for recreation facilities which revealed some positive associations (Table S1). Features of communal space, land use and density predominantly had non-significant effect on overall physical activity. There was some evidence for a relationship between pollution (perceived sewage and air pollution and audited noise pollution) and overall activity: pollution was negatively related to overall physical activity and overall leisure-time physical activity (Florindo et al., 2013, Van Lenthe et al., 2005) but positively related to overall active travel (Van Lenthe et al., 2005). In addition, the presence of physical activity and health clubs and facilities predominantly had a positive association with overall physical activity while there was a null effect of walking or cycling trails apart from two studies (Adlakha et al., 2015, Eichinger et al., 2015) reporting a positive effect with overall leisure-time physical activity and in one of the studies overall active travel (Adlakha et al., 2015), but not overall physical activity. The only study investigating overall physical activity in a sample residing in China showed that, as reported elsewhere for walking outcomes (Jack and McCormack, 2014, King et al., 2006), connectivity was differentially related to overall active travel and leisure-time activity, demonstrating only a negative association with overall leisure-time physical activity (Zhou et al., 2013).

### 3.2 Independent social environment correlates

When simultaneously accounting for aspects of the physical environment, cohesion (social cohesion and sense of belonging) and external neighbourhood reputation overall had a positive relationship with walking (Supplementary material: Table S2). Internal neighbourhood reputation (sense of progress in your neighbourhood) and a composite measure of social capital (assessing multiple dimensions including cohesion, reciprocity and trust) had a negative association with walking and leisure-time walking (Caspi et al., 2013, Mason et al., 2011). In contrast, the composite measure was positively related to active travel by walking (Caspi et al., 2013). There were predominantly null associations with walking and crime and inconsistent findings for social networks, safety and composite measures of cues of social disorder (including cues such as adults loitering, presence of police and people drinking alcohol openly) and trust and engagement. Studies revealed more consistent evidence for a relationship between social networks and MVPA. Individual studies found positive relationships between trust, engagement and a composite measure of social capital and MVPA, although the evidence was limited by the paucity of research. There was some evidence for a relationship between crime and MVPA but little evidence for an association between safety and cues of social disorder and MVPA (Table S2).

There was also a lack of evidence for an association between perceived and objectively-measured crime and overall physical activity. However, 6 of 9 studies reported a significant relationship between perceived safety and overall physical activity, most of which were in the expected direction. Seven studies exploring individual (rather than composite) social capital variables and overall activity revealed inconsistent evidence that social cohesion (Cleland et al., 2010, Eichinger et al., 2015, King, 2008, Li and Fisher, 2004), sense of belonging (Prince et al., 2011, Prince et al., 2012) or engagement (Prince et al., 2011, Prince et al., 2012, Poortinga, 2006) were related to activity and presented only null findings for social networks (Bird et al., 2009, Poortinga, 2006).

### 3.3 Neighbourhood-based physical activity

Seven studies used neighbourhood-based physical activity as a primary outcome, of which 3 examined walking (Fisher et al., 2004, Jack and McCormack, 2014, Mason et al., 2011), 2 examined MVPA (Handy, Cao & Mokhtarian, 2088; Karusisi, Bean, Oppert, Pannier & Chaix, 2012) and 2 explored overall physical activity (King, 2008, Li and Fisher, 2004). Although there was more consistent evidence for correlates at the level of variable clusters (e.g. shared space), there were too few studies exploring the same environmental variables to draw reliable conclusions.

### 3.4 Multiple and interactive environmental influences

More models showed both social and environmental correlates than one level of correlates or none (Table 3, percentage of models revealing both social and environmental correlates: walking: 44% of models; MVPA: 53% of models; overall physical activity: 33% of models). Studies presented fewer models that had only physical correlates, only social correlates or neither social nor physical correlates. The only exception was overall physical activity for which studies included an equal number of models including both social and physical correlates and physical correlates only. The majority of models which included an interaction term found an interactive or modifying effect of physical and social correlates on the physical activity outcome. However, there were only 8 models which included an interaction term. These results suggest multiple and interacting levels of environmental influence on physical activity.

**Table 3:** Significance of physical and social correlates across models with different physical activity outcomes.

**Significant correlates**	**Walking N (% of models)**	**MVPA N (% of models)**	**Overall PA N (% of models)**	**Total N (% models)**
Both physical and social	11 (44.0)	10 (52.6)	10 (33.3)	28 (43.1)
Physical only	8 (32.0)	4 (21.1)	10 (33.3)	19 (29.2)
Social only	2 (8.0)	1 (5.3)	3 (10.0)	5 (7.7)
Neither	4 (16.0)	4 (21.1)	7 (23.3)	13 (20.0)
Interaction^a^	4 (66.7)	0 (0.0)	1 (100.0)	5 (62.5)

a Interaction terms were included for 8 models with walking (n=6), MVPA (n=1) and overall PA (n=1) as outcomes. The denominator used to calculate percentages for ‘both physical and social’, ‘physical only’, ‘social only’ and ‘neither’ rows is the number of models for each physical activity outcome. The denominator used to calculate percentages for the ‘interaction’ row is the number of models with interaction terms for each physical activity outcome.

All studies exploring interactive effects of physical and social environment variables and physical activity examined an intervening role of crime or safety (Bracy et al., 2014, Jack and McCormack, 2014, King, 2008, Van Dyck et al., 2013). Two of these found an interaction between perceived crime and walkability (measured using multiple variables, including street connectivity, destination density and transit-stop access). In one study, participants’ perception of crime was lower in neighbourhoods that were objectively highly walkable, yet active travel (walking only) significantly decreased when participants’ perceptions of crime were higher in neighbourhoods with high walkability but not in neighbourhoods with mid or low walkability (Jack & McCormack, 2014). Bracy et al. (2014) presented only a small number of significant interactive effects of physical environment variables and perceived safety on objectively-measured MVPA, compared with a larger number of insignificant interaction terms. One significant interaction was between walkability and crime: participants who lived in highly-walkable neighbourhoods and perceived low levels of crime performed an additional 91.2 min of MVPA/week than participants living in neighbourhoods with low walkability and perceiving low levels of crime. There was only 38.8 min difference in MVPA/week (a significantly smaller difference) between participants living in neighbourhoods of high or low walkability and perceiving high levels of crime. Van Dyck et al. (2013) also reported that social cohesion and perceived safety partly mediated the effect of objectively-measured connectivity and destination density (combined in a single metric encompassing connectivity of streets and food outlets, supermarkets, physical activity facilities and playgrounds) on self-reported leisure-time walking but not walking for active travel in women living in deprived neighbourhoods in Australia. Social cohesion explained 13.3% of the association between the connectivity and destination density metric and leisure-time walking, while safety explained 20.0% of the association, suppressing the effect of the physical metric.

In a sample of 645 adults in Denver, USA, crime mediated the relationship between yard maintenance and overall neighbourhood-based physical activity (King, 2008). Sobel’s tests of mediation also found that associations between physical activity and: yard maintenance, window bars and litter operated in part through social cohesion. The significance of the relationship between physical activity and yard maintenance and window bars was lost when social environment variables were included in analyses.

## 4 Discussion

From the 46 studies identified that simultaneously examined neighbourhood physical and social environment correlates of physical activity, there was limited evidence for consistent, independent physical and social correlates in terms of specific variables. There was some support for a positive association between physical activity facilities and both walking and overall physical activity, and weaker evidence for a positive relationship between walking and both high quality communal spaces and good street conditions. Active travel and leisure-time physical activity appeared to have differential relationships with the physical environment in terms of presence or direction of an association with perceived access to service, connectivity and pollution. These results support domain-specificity in ecological models of physical activity (Sallis et al., 2006). There was some evidence for increased physical activity in individuals reporting higher levels of social cohesion and a sense of belonging to the neighbourhood. Although few consistent specific correlates were identified, studies tended to report both significant physical and social correlates in models, rather than only physical or social correlates, or neither. This finding is supportive of ecological models of physical activity which posit multiple levels of environmental influence on activity. Studies tended to examine fewer social environment variables than physical environment variables and there were very few studies examining interactive effects.

In terms of social environment variables, most studies examined cohesion and safety related variables, finding a positive relationship whereby participants living in socially-cohesive neighbourhoods engaged in more walking, MVPA and overall physical activity but more inconsistent findings for safety and crime. Interestingly, the only studies investigating the effects of trust and participation in organisations or activities (engagement) on walking, found conflicting results (Mason et al., 2011, Poortinga, 2006). Both studies used single-item measures of trust and participation in the UK but while one study was nationwide sample of private household owners (Poortinga, 2006) the other was a sample of adults living in income-deprived neighbourhoods (Mason et al., 2011). Mason et al. (2011) suggested unexpected negative associations between walking and trust and participation could be due to reverse causality, where individuals living in income-deprived neighbourhoods who do not regularly walk in their neighbourhood are less familiar with the negative social aspects and therefore have higher levels of trust. This highlights a general need to assess the direction of causality which is not possible in cross-sectional analyses. Inconsistent findings regarding the relationship between crime, safety and physical activity were not unexpected and have been demonstrated previously (Foster & Giles-Corti, 2008).

Recent systematic reviews separately highlight associations between active living and the physical environment (Astell-Burt et al., 2014, McCormack and Shiell, 2011) and social environment (Samuel et al., 2014). Despite this, null associations and inconsistent findings were frequently reported. Moreover, it is possible that null associations were under-reported, as often variables with an insignificant effect in univariate models are not included in multivariate models and therefore would not reported in this review. Inconsistent and null results for physical correlates may also be partly attributable to only including studies that simultaneously included social environment correlates in statistical models. However, it is problematic to frame this finding wholly in terms of the relative importance of social and physical environmental correlates of physical activity: studies tended to examine many more physical correlates than social correlates, potentially leading to problems around colinearity or over-adjustment, and the adjustment for social correlates was not standardised (in number or type of social correlates) across studies. Null associations could also arise from methodological limitations that inhibit identification of environmental correlates; such limitations could be amplified by the complexity of the relationship between the environment and physical activity. This review highlighted several such methodological limitations in the literature that future research should try to ameliorate.

Firstly, a lack of sensitivity and specificity could obscure real associations. Physical activity that is neighbourhood-based could be expected to relate more closely to environmental features of the neighbourhood. Optimising the correspondence of neighbourhood boundaries across exposures and outcomes is likely to be advantageous in heightening sensitivity to detect hypothesised relationships. Providing participants with clearly defined neighbourhood boundaries or using guidelines such as a 5–10 min walk from the participants’ residence could be useful where appropriate to the research question and measures (Smith, Gidlow, Davey & Foster, 2010). Pairing Global Positioning System (GPS) data with accelerometry data also presents an opportunity to objectively operationalise neighbourhoods as an ‘activity space’, for which social and physical environment data could closely correspond (Boruff, Nathan & Nijënstein, 2012). Only 7 of 46 studies used neighbourhood-based physical activity in this review; there is scope for further research using this outcome.

Specificity is also valuable in terms of operationalization of variables and the conceputalisation of salient environmental correlates in different contexts and population groups. In a sample of 190 older adults in the USA, univariate analyses demonstrated a significant association between physical activity and the presence of window bars but not neighbourhood-watch signs (King, 2008). This distinction demonstrates the importance of specific operationalisation: two forms of physical forms of security measures could differently affect behaviour by representing either collective or individualistic approach to neighbourhood security. While window bars protect individual houses, neighbourhood-watch signage implies a community effort for protection through a commitment to collective surveillance.

Future research would also greatly benefit from context- and group-specific conceptualisation of environmental influences. Wen, Kandula, & Lauderdale (2007) highlighted that the strength of neighbourhood social and physical influences varied across racial and ethnic groups in a sample of White, Black, Hispanic and Asian adults in California, USA. The effect of the environment may also vary across neighbourhood-level deprivation. Van Dyck et al. (2013) found that the effect of objectively-measured connectivity and destination density on leisure-time walking was partially mediated by perceived physical aesthetics, safety and social cohesion. The authors suggest that in socioeconomically-deprived contexts, perceived micro-scale features (e.g. aesthetics) and social environmental features may override structural features that would create ostensibly ‘walkable’ neighbourhoods.

Secondly, this review underlined a need for study methodologies to apply conceptualisations of environmental variables as having direct or indirect influences on physical activity and use appropriate statistical analyses to test these conceptual hypotheses. Preliminary evidence was presented that walkability and perceived safety may have an interactive effect on physical activity. Interestingly, while two studies reported an interaction between walkability and perceived safety, they appeared to have different effects on activity (Bracy et al., 2014, Jack and McCormack, 2014). This could be partly due to neighbourhood contexts and differences in street layout and urban form between cities in the USA and Canada. Nevertheless, both studies demonstrated that walkability was particularly important for activity when participants perceived high levels of neighbourhood crime. In addition, mediation analyses by King (2008) found that perceived levels of crime mediated the association between overall community-based physical activity and yard maintenance and window bars, rendering a direct effect of these environmental variables insignificant. Likewise, Van Dyck et al. (2013) reported that the relationship between a combined metric of connectivity and destination density and leisure-time walking was mediated by social cohesion and perceived safety in a sample of women in socioeconomically deprived neighbourhoods. Kaczynski & Glover (2012) also demonstrated interactive effects of the physical and social environment on physical activity, although findings were not included in this review as main effects were not presented. In a study of 380 adults in Canada, they found that higher levels of recreational walking were reported for participants living in highly socially-connected neighbourhoods (combined assessment of cohesion and trust) while higher levels of walking for active travel were reported for participants living in highly walkable neighbourhoods. Highest levels of walking for recreation or travel were in neighbourhoods with both high walkability and social connectedness. Furthermore, a mediating effect of crime on the association between recreational facilities and self-reported MVPA was found in a sample of 781 adults living in Chicago, USA (Berchuck et al., 2016). Interestingly, the suppression effect of crime was only apparent in neighbourhoods in the south of the city – which historically have higher rates of crime and poverty and a larger ethnic minority population than neighbourhoods in the north - highlighting the context-specificity of the relationship. This study was not included in the review as it did not test the independent association between crime and MVPA.

Together these findings demonstrate the benefit of simultaneous analysis in elucidating potential pathways between the environment and activity, by ensuring that significant relationships are not obscured by unaccounted for aspects of the environment. However, few studies used interaction or mediation analyses to explore hypotheses arising from ecological models positing that social and physical environmental variables work together to affect physical activity. This review supplies evidence of the current lack of research exploring interactive environmental effects on physical activity in adults and therefore provides support to previous calls by researchers in the field to make this a future research priority (Gubbels, Van Kann, de Vries, Thijs, & Kremers, 2014).

There are several limitations to this review. A meta-analysis was not possible owing to the heterogeneity between studies; this may be possible at a later stage as the evidence base grows. Although this review could assess the relationship between physical environment variables and physical activity while accounting for social environment variables, and vice versa, the environmental variables that were accounted for in models varied between studies therefore it was not possible to draw conclusions regarding the comparative importance of social and physical environment correlates. This is an unavoidable limitation but should be acknowledged when interpreting the results of this review.

### 4.1 Conclusions

Results drawn from 46 studies revealed an inconsistent evidence base for environmental correlates of physical activity, with some support for specific physical and social environment correlates and support for multiple levels of environmental influence on activity. Further research is needed to substantiate reported findings. The heterogeneity of physical environmental measures and non-standardised consideration of social environmental constructs could contribute to inconsistent findings in the literature and should be considered when interpreting presented findings. Interaction or mediation analysis will be valuable in exploring potential pathways between the environment and activity and conceptualising environmental correlates in terms of their direct or indirect effect on physical activity. Resolving additional methodological issues in future research may also elucidate complex relationships and thereby map key environmental correlates of physical activity.

## Conflicts of interest

The authors declare no financial, personal or other conflicts of interest.
