# Supplementary material for: Simultaneous evaluation of physical and social environmental correlates of physical activity in adults: A systematic review
Source: SSM Popul Health. 2017 May 15;3:506–15. doi: 10.1016/j.ssmph.2017.05.008 (PMC5769071; doi:10.1016/j.ssmph.2017.05.008)
Supplement: Supplementary file 1 — Supplementary material [file mmc1.docx]

**Supplementary material**

**Table S1:** Results for physical environment variables

|  |  | Walking & Light PA | | | MVPA | | | Overall PA | | |
| --- | --- | --- | --- | --- | --- | --- | --- | --- | --- | --- |
|  | Studies(N) | Negative | Null | Positive | Negative | Null | Positive | Negative | Null | Positive |
| COMMUNAL SPACE | |  | | |  | |  |  | | |
| Green/open space | 12 |  | 3^†^ | **2** |  | 2 | **2^†^** |  | 6^†^ | **2** |
| General design | 4 |  |  |  |  |  | **1^†^** |  | 5 | **2^†^** |
| Aesthetics | 10 |  | 5 | **1^†^** | **1** | 3 | **2** |  | 6^†^ | **2** |
| Cues of disorder | 6 |  | 4 |  |  | 2 |  | **1^†^** | 2 | **2** |
| FACILITIES/AMENITIES | |  | | |  | | |  | | |
| Recreation facilities | 14 | **1^†^** | 4 | **4^†^** |  | 4 | **4^†^** |  | 5 | **3** |
| Food outlets | 6 |  | 1 |  |  |  | **1^†^** |  | 5^†^ | **1^†^** |
| Institutions | 3 |  | 1 |  |  | 1 |  |  |  | **1** |
| Shops | 8 |  | 2**^†^** | **2** |  | 1 | **2** |  | 4 | **2** |
| WCs | 1 |  | 1 |  |  | 1 |  |  | 1 |  |
| Services | 4 | **1^†^** | 1 | **4^†^** |  | 1 |  |  |  | **1** |
| STREET CONDITIONS | |  | | |  | | |  | | |
| Pavements | 13 | **1** | 2 | **1** |  | 5 | **1** | **1** | 7 | **2^†^** |
| Pedestrian envir | 8 |  | 3 | **2** |  | 1 | **2^†^** |  | 5 |  |
| Street lighting | 8 |  |  |  |  | 4 |  |  | 5 | **1** |
| Traffic | 17 |  | 6 |  |  | 5 | **2** | **2^†^** | 11 |  |
| Pollution | 3 |  |  |  |  |  |  | **2** | 2 | **1^†^** |
| PA FACILITIES | |  | |  |  |  |  |  |  |  |
| PA/health clubs/facs | 13 |  | 1 |  |  | 5 | **2** |  | 1 | **3** |
| Walk/bike trails | 10 |  | 3 | **1** |  | 2 |  |  | 10 | **3** |
| LAND USE |  |  | |  |  |  |  |  |  |  |
| Density | 7 |  | 2 |  |  | 3 | **1** |  | 3**^†^** |  |
| Land use mix | 5 | **1** |  | **2** |  | 3 | **2^†^** |  | 2 | **1** |
| Resid’l density | 4 | **1** | 2 | **1** |  | 3 | **1** |  | 1 | **1** |
| CONNECTIVITY | |  |  |  |  |  |  |  |  |  |
| Connectivity | 7 |  | 2 | **2^†^** |  | 4 | **2^†^** | **2^†^** | 2 | **1^†^** |
| Connectivity + density | 1 | **1** |  | **1** |  |  |  |  |  |  |
| Transit | 5 |  | 1 |  |  | 4 | **1** |  | 4 |  |

† Conflicting results; institutions were defined as public, financial, educational, social or religious organisations

**Table S2:** Results for social environment variables

|  |  | Walking & Light PA | | | MVPA | | | Overall PA | | |
| --- | --- | --- | --- | --- | --- | --- | --- | --- | --- | --- |
|  | Studies(N) | Negative | Null | Positive | Negative | Null | Positive | Negative | Null | Positive |
| SOCIAL CAPITAL | |  | | |  | | |  | | |
| Capital | 2 | **1^†^** |  | **1^†^** |  |  | **1^†^** |  |  |  |
| REPUTATION |  |  |  |  |  |  |  |  |  |  |
| External reputation | 1 |  |  | **1** |  |  |  |  |  |  |
| Sense of progress | 1 | **1** |  |  |  |  |  |  |  |  |
| SOCIAL NETWORK | |  |  |  |  |  |  |  |  |  |
| Networks | 7 | **1** | 2 | **1** | **1** |  | **4** |  | 2 |  |
| TRUST & EMPOWERMENT | |  |  |  |  |  |  |  |  |  |
| Trust | 2 | **1** |  | **1** |  |  | **1** |  | 1 |  |
| Engagement | 4 | **1** |  | **1** |  |  | **1** |  | 2 | **1** |
| COHESION & SAFETY | |  | | |  | | |  | | |
| Cohesion | 11 |  | 2 | **4** |  | 1 | **2** |  | 5**^†^** | **2** |
| Belonging | 5 |  |  | **4** |  |  | **1** |  | 1 | **1^†^** |
| Crime | 22 | **2^†^** | 7 |  | **6^†^** | 7^†^ |  | **3** | 12^†^ | **1** |
| Safety | 18 | **1** | 6**^†^** | **5^†^** |  | 1 |  | **2^†^** | 4 | **5^†^** |
| Disorder | 7 | **2** | 2 | **1** | **1** | 5 |  |  | 1 |  |

† Conflicting results.
